# Microwave Sintering of SiAlON Ceramics with TiN Addition

**DOI:** 10.3390/ma12081345

**Published:** 2019-04-25

**Authors:** Özgür Sevgi Canarslan, Roberto Rosa, Levent Köroğlu, Erhan Ayas, Alpagut Kara, Paolo Veronesi

**Affiliations:** 1 Department of Materials Science and Engineering, Eskişehir Technical University, 26555 Eskişehir, Turkey; leventkoroglu@eskisehir.edu.tr (L.K.); erayas@eskisehir.edu.tr (E.A.); akara@eskisehir.edu.tr (A.K.); 2 Department of Engineering “Enzo Ferrari”, University of Modena and Reggio Emilia, 41125 Modena, Italy; roberto.rosa@unimore.it (R.R.); paolo.veronesi@unimore.it (P.V.)

**Keywords:** SiAlON composites, microwave sintering, 2.45 GHz, single mode cavity, susceptor

## Abstract

α-β SiAlON/TiN composites with nominal composition of α:β = 25:75 were fabricated by microwave sintering. The effect of titanium nitride addition on the phases, microstructure, microwave absorption ability and mechanical properties (Vickers hardness and fracture toughness) of the SiAlON-based composites were studied. Finite Difference Time Domain (FDTD) software was used for the numerical simulation in order to assess the most suitable experimental setup. Sintering trials were performed in a single mode microwave furnace operating at 2.45 GHz and a power output of 660 W, for a reaction time of 30 min. SiC blocks were used as a susceptor to accelerate the microwave processing by hybrid heating, with reduced heat losses from the surface of the material of the α-β SiAlON/TiN composites. The optimum comprehensive mechanical properties, corresponding to a relative density of 96%, Vickers hardness of 12.98 ± 1.81 GPa and Vickers indentation fracture toughness of 5.52 ± 0.71 MPa.m^1/2^ were obtained at 850 °C when the content of TiN was 5 wt.%.

## 1. Introduction

SiAlONs are ceramics named after the elements they contain: silicon (Si), aluminium (Al), oxygen (O) and nitrogen (N). They are a large family of the so-called solid solutions based on silicon nitride (Si_3_N_4_), where Si–N bonds are partly replaced with Al–N and Al–O bonds. SiAlON ceramics are widely used for a large range of structural and engineering applications such as gas turbines, heat insulators, cutting tools and spray nozzles and as refractories in the molten metal industry thanks to their excellent mechanical properties, such as high strength, high decomposition temperature, excellent thermal shock and good wear-resistance, low coefficient of friction and resistance to corrosive environments [1,2]. Such properties are closely linked to the microstructure, chemical composition and fabrication process. SiAlON-based ceramics typically exhibit one of two structures, the growth of which is dependent on their general chemical formula. The first of these is α-SiAlON, which has a formula of MxvSi_12−(m+n)_Al_m+n_O_n_N_16−n_ (x = m/v; v is the valence of the metal, and M represents Li, Mg, Ca, Y, or a rare earth metal) and generally exhibits equiaxed grains. The other one is β-SiAlON, or Si_6−z_Al_z_O_z_N_8−z_ where z denotes the number of Si-N bonds substituted by the Al-O bonds (0 < z ≤ 4.2) [1,2,3], which forms elongated grains. The difference in grain structure is responsible for the higher Vickers hardness and resistance to oxidation and erosion of α-SiAlON, while β-SiAlON has greater strength and toughness. This has driven a recent growth in interest in mixed α/β-SiAlON materials to achieve a combination of favorable characteristics [2,4]. 

The most effective method of increasing toughness of SiAlON’s is through the use of a second-phase reinforcement in order to generate a sialon matrix composite [5]. In composite form, outstanding improvements can be achieved such as increased fracture toughness, reduced strength variability, reduced flaw sensitivity, reduced crack propagation and better reliability; and even more significantly, the failure manner of SiAlON composites can be changed and controlled. Composites of silicon nitride and titanium nitride have been widely investigated in literature, demonstrating the possibility to obtain a combination of high hardness, high strength and good fracture toughness values [6,7,8,9].

Recently, there have been many studies on the use of microwaves to process ceramic materials. The main advantages of using microwave energy to sinter ceramic materials compared to conventional techniques are: reduced sintering time and lower sintering temperature; early formation of rounded porosity; improved diffusion by means of ponderomotive forces, leading to an improved capacity to produce unique microstructures, which typically could not be achieved by other conventional methods [10]. Microwave processing of ceramics has gained significant attention over the last few decades due to its superiority over conventional processing methods in areas such as volumetric heating, faster processing and lower energy consumption. Since sintering of the silicon nitride based composites involves a high temperature operation, often exceeding 1600–1750 °C [11], there is high interest in a technique able to decrease such temperatures and to speed up the process. However, the presence of other materials during microwave sintering, like the insulating materials and possible use of susceptors, plays a critical role in the successful microwave processing of this low lossceramic [12,13,14,15,16,17]. In particular, susceptor-assisted microwave heating (also referred as hybrid microwave heating) of ceramic materials is relevant to SiAlON processing because it allows to improve the coupling of microwaves at low temperature with a low loss ceramic by either using high loss external susceptors or adding lossy conductive or magnetic materials in the form of secondary phases [18]. Silicon nitride, in its pure form, is a poor microwave absorber due to its low dielectric loss at room temperature [19] but it can be proficiently microwave-heated above 1300 °C, by selective coupling of microwaves to the grain boundary glassy phases [19]. 

Although there has been considerable research in the field of SiAlON-TiN composites, there is a dearth of literature addressing the use of microwave energy to sinter α/β-SiAlON-TiN composites. In particular, addition of TiN is expected to improve the coupling of microwaves [20], as well as to increase both the mechanical and electrical properties of SiAlON. Therefore, the purpose of the present work is the low temperature sintering of α/β-SiAlON–TiN composites with SiC susceptor through 2.45 GHz microwave energy in single mode cavity and to investigate dielectric properties, phase transformation, microstructure development, hardness and electrical properties. The novelty of this work lies in the use of Field Assisted Sintering Techniques (FAST) different from Spark Plasma Sintering (SPS), and in particular of microwave heating at 2.45 GHz in predominant electric field regions, to process SiAlON matrix composites. The aim is to be able to exploit the microwave selective heating occurring in the liquid phase at the grain boundary, which is produced during sintering, to reduce overall processing temperatures.

## 2. Materials and Method

### 2.1. Preparation of Powder Mixture

**Figure 1:**
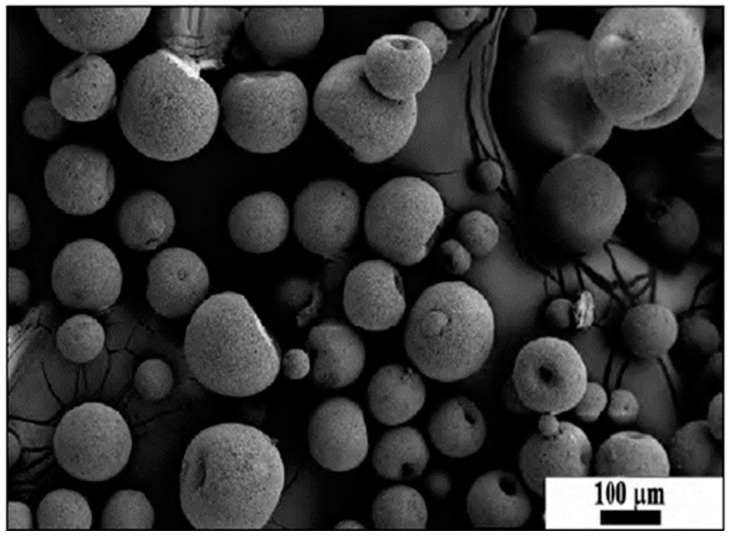
Scanning electron microscopy (SEM) image of the spray dried SiAlON granules.

Spray dried SiAlON granules were supplied by MDA Advanced Ceramics Ltd. (Eskisehir, Turkey) and are shown in Figure 1. The composition was designed to provide 25% α-SiAlON/75% β-SiAlON in the final product. In order to prepare α–β SiAlON composition (designated as SN), 89.4 wt.% Si_3_N_4_ (SN-E10, UBE Industries Ltd., Tokyo, Japan), 5.4 wt.% AlN (HC Starck GmbH, Munich, Germany), 2.5 wt.% Al_2_O_3_ (Sumitomo, Osaka, Japan), 4.73 wt.% Y_2_O_3_ (Shin Etsu Chemical Co., Ltd., Tokyo, Japan), 0.4 wt.% Sm_2_O_3_ (Sigma-Aldrich, Darmstadt, Germany) and 0.12 wt.% CaCO_3_ (Riedel-de Haën, Seelze, Germany), were used as starting powders. The powders were mixed through attrition milling with de-ionized water as liquid media and Si_3_N_4_ grinding balls for 2 h in a polyamide container. The slurry was then dried in a spray drier (Nubilosa, Konstanz, Germany) to obtain spherical granules of around 100 μm in diameter, under suitable conditions [21]. SiAlON granules were incorporated with various amounts of TiN from 2.5 to 5 wt.% by mixing with ZrO_2_ balls in isopropyl alcohol media for 1.5 hr at 350 rpm in a Retsch GmbH PM100 planetary ball mill (Retsch GmbH, Haan, Germany).

### 2.2. Microwave Sintering

Green disc-shaped samples of 10 mm diameter were obtained by uniaxial pressing at 200 MPa in order to give strength to specimens before microwave sintering. The resulting green density at these pressure levels was almost identical, with measured values of 57.0% ± 1% in the case of 2.5% TiN addition and 57.2% ± 1% in the case of 5% TiN addition.

**Figure 2:**
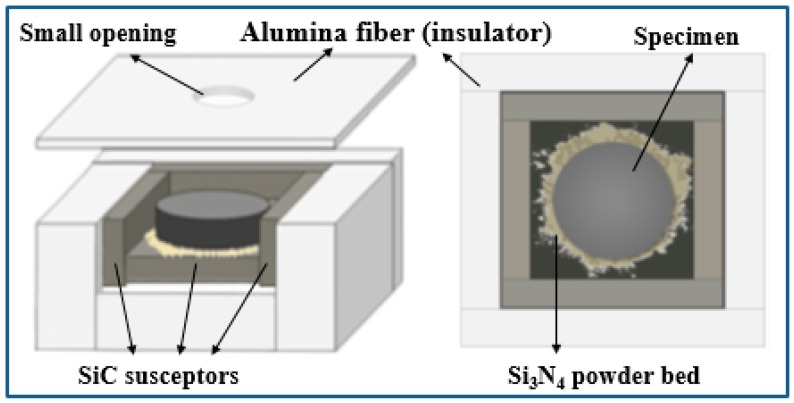
Illustration of the crucible configuration used in the experiments.

During microwave sintering, it is quite important to select a low dielectric loss refractory material, thus, alumina fiber was chosen as a crucible due to its high melting temperature (1870 °C) and excellent thermal shock resistance. The dielectric loss of the alumina fiber board at 1450 °C was 0.025 at 2.45 GHz, which ensures minimum absorption of microwave energy. This type of crucible acts as a good insulator and it is transparent to microwaves; thus, it does not affect the field inside the microwave applicator. The configuration used in the microwave sintering trials is shown in Figure 2.

**Figure 3:**
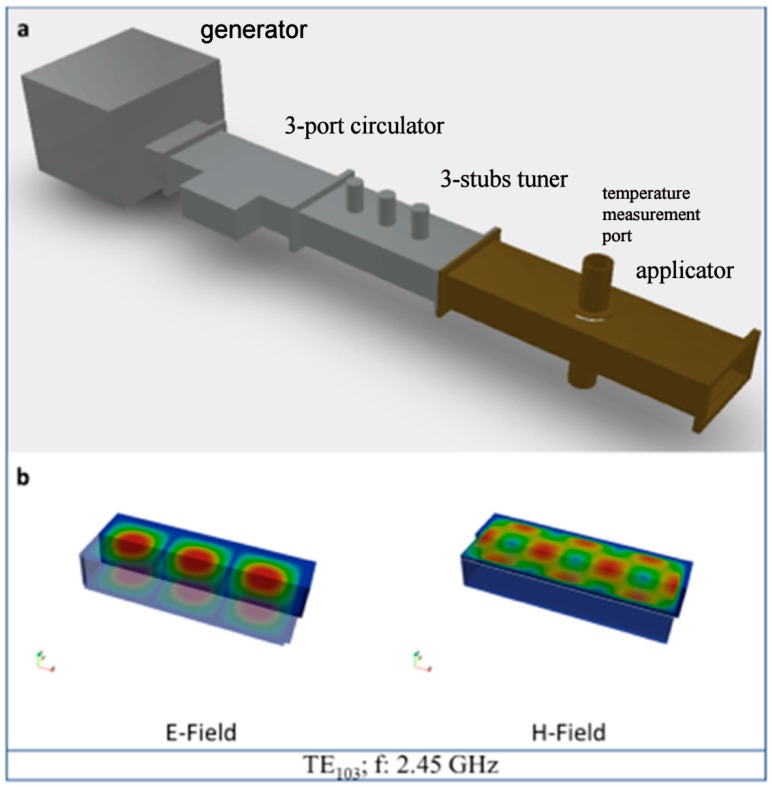
(**a**) Schematic of the single mode furnace, with removed short circuit and (**b**) simulated E- and H fields in the empty applicator. The applicator dimensions are 86 x 43 x 185.6 mm.

The samples were placed, one per run, inside a lining made of alumina fiber (Zircar Buster A-15), surrounded by SiC blocks acting as auxiliary microwave absorbers (susceptors). A Si_3_N_4_ powder bed was used at the interface between the sample and the auxiliary absorber in order to avoid the reaction between the pellet and the SiC block. Such load was inserted in the region of predominant electric field of a TE_103_ single mode 2.45 GHz microwave furnace, shown in Figure 3a. The single mode microwave applicator operates at a center frequency of 2.45 GHz ISM (allocated for Industrial, Scientific and Medical use) frequency. It consists of a 3 kW generator (magnetron, TM030, MKS Alter, Reggio Emilia, Italy), a 3-stub tuner used for impedance matching, a 3-port circulator with reflected power measurement on the third port, connected to a water load, a transmission line (WR340 waveguide), a tunable applicator and a dedicated control system, as shown in Figure 3b. The magnetron output power was set to 660 W.

Numerical simulation was employed prior to experimental activity to assess the most suitable setup for rapid and controllable heating of the load to the desired temperature. Numerical simulation of the microwave heating of the sample was performed using the FDTD (Finite-Difference Time-Domain method) software Quickwave 2018 (QWED Sp. z.o.o., Warsaw, Poland) considering two loading conditions; i.e., with or without the SiC auxiliary absorber. Both scenarios were used to investigate the possible heat generation starting from room temperature and in the case of pre-heating of the load at 1000 °C. The latter option addresses the fact that the pressed powders are a low loss material at room temperature, while their loss factor dramatically increases as the temperature increases [22,23,24].

**Figure 4:**
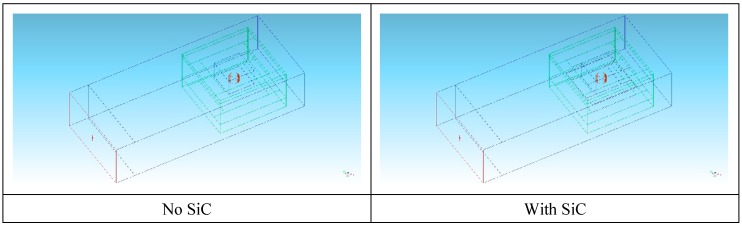
Model of the loaded applicator without or with SiC susceptors: green = alumina fiber lining; black = SiC elements; red = pressed powders. Microwave input is from the left port (in red).

Figure 4 shows the geometry of the model, where the microwave generator was simulated considering the WR340 waveguide fed from one end, in the TE_10_ mode. 

Table 1 summarizes the dielectric and thermal properties used in the model at the indicated temperature [23,24,25,26,27,28].

**Figure 5:**
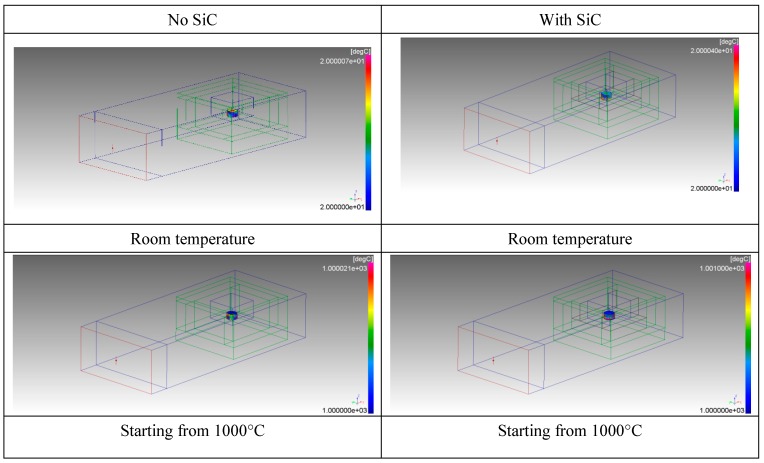
Temperature distribution in the pressed powders after 10 seconds of microwave heating starting from room temperature or 1000 °C, without SiC absorbers or with SiC absorbers.

The walls of the applicator were set as perfectly electric conductors and in this simplified model no density variations were taken into account, as the aim was the identification of the most suitable experimental conditions to perform microwave heating. The Basic Heating Module (BHM) of the software Quickwave was used to estimate the load temperature, neglecting air convection inside the alumina lining and radiation losses. Simulation results are summarized in Figure 5, for the two scenarios with and without SiC.

**Figure 6:**
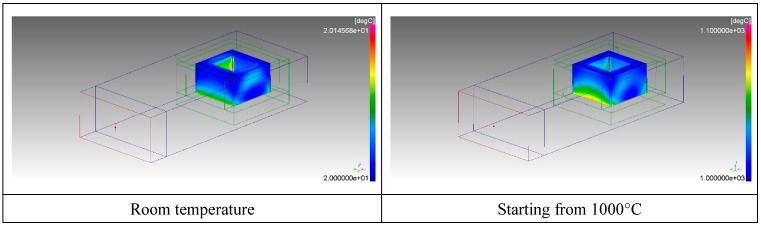
Temperature distribution in the SiC absorbers after 10 seconds of microwave heating starting from room temperature or 1000 °C.

The results indicated that heat generation occurs in the center of the pressed powders, when heated starting from room temperature, but to a lesser extent in the case of SiC used as a co-absorber. This expected result is due to the available microwave power split, also on the SiC element, whose heating after 10 seconds is not enough to affect the pressed powder temperature rise. This is more evident when also plotting the temperature distribution inside the SiC blocks, as shown in Figure 6.

The use of SiC results in a much higher load temperature, mainly due to conduction of the heat generated in the bottom SiC plate. The resulting simulated temperature of the pressed powders, at 1000 °C, was much lower than the SiC one, despite the increase of the loss factor of the former. This is due to the higher density (and hence, mass) and specific heat of the pressed powders compared to SiC. As a consequence, despite the higher power density in the pressed powders compared to SiC, a lower temperature increase is achieved due to their higher heat capacity. Given these premises, the experimental setup chosen is the one depicted in Figure 2, with the SiC susceptor. 

**Figure 7:**
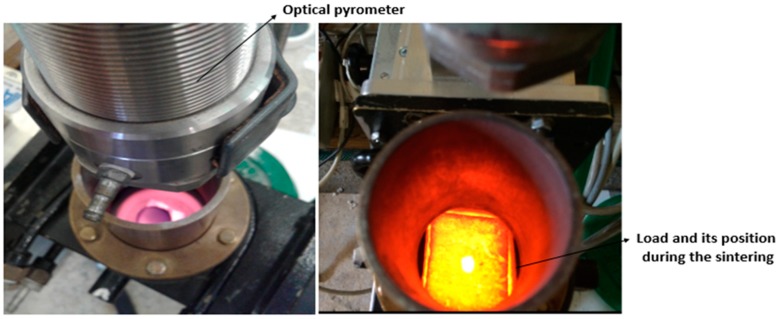
The top view of the load and its position in the furnace during microwave processing.

In order to monitor the temperature during processing, the alumina lining has a small opening at the top (3 mm diameter) to allow non-contact temperature measurement by an optical pyrometer (Sitel, Lissone, Italy), as shown in Figure 7. Such temperature measurement technique provides only the surface temperature of the sample, hence it can be an underestimate of the temperature existing at the sample-SiC plate interface, as shown by the modeling results of Figure 5. Modeling results of the complete heating cycle show that at a surface temperature of 850 °C, the bottom temperature of the sample is in excess of 900 °C. However, this temperature difference was experimentally reduced introducing an isothermal step at the maximum temperature, by reducing the forward microwave power.

The silicon carbide blocks are used to absorb microwaves and to develop heat in the first stages of the heating process, when the dielectric properties of the samples alone do not allow fast and effective heating. Si_3_N_4_ based ceramics without using any high lossy additives are relatively transparent to microwaves and poor couplers at room temperature. Once the sample, heated by the silicon carbide block, reaches a temperature corresponding to higher dielectric losses, it starts coupling with the microwaves to a larger extent and the power transfer from the electromagnetic field proceeds more rapidly, leading to sintering or, eventually, melting. However, the sample integrity strictly depends on the temperature gradient, imposing an upper limit to how fast the load can be heated up. The final step of the heating treatment consisted of 10 min air cooling by natural convection outside the applicator. The procedure was repeated on four different sets of samples to verify its reproducibility.

The whole heating cycle can be potentially divided into three stages: constant heating (0–500 °C), rapid heating up to the maximum achievable temperature given by the used setup and final isothermal treatment at the maximum temperature (780 or 850 °C). Such maximum temperature is achieved as a balance of heat losses through the alumina lining and the heat generated in the load. Hence, lossier loads are expected to reach higher temperatures in this setup. Composition and sintering temperature of the samples were varied according to Table 2:

The relative density of the samples was measured by the Archimedes method within the distilled water medium. Vickers hardness (HV_20_) from the polished surfaces of the sintered samples was measured by using an indenter (Emco-Test Prüfmaschinen, GmbH, Kuchl, Austria) with a load of 20 kg. The Vickers indentation fracture toughness (VIF) of the samples was evaluated from the radial cracks formed during the indentation test using the formula developed by Evans and Charles given below [29].

(1) $$VIF=\frac{0.15k\;{\left(\frac{c}{a}\right)}^{-\frac{3}{2}\;}Hv20\sqrt{a}}{\Phi }\;\left(MPa.m^{\frac{1}{2}}\right),$$

where *VIF*, *Hv* are the Vickers indentation fracture toughness and hardness of the material; *a* and *c* are the impression radius and the crack length in the diagonal direction of the indent, Φ and *k* are the constraint factor (Φ = 3) and the surface constant (*k* = 2.6). Noticeably, this measurement method is quite debated in the literature, and this is reflected by the numerous different equations which have been developed to estimate fracture resistance by this technique [30]. The VIF test is here used to compare the behavior of the sintered specimens when locally loaded by a Vickers indenter provoking multiple cracks, under 3D stress state. At least three measurements were repeated for each sample.

To perform the microstructure analysis, sample surfaces were polished and coated with Au-Pd alloy. By using the back scattered electron detector, the microstructure of the cross sections was observed by scanning electron microscopy (SEM, Supra 50vp, Carl Zeiss, Oberkochen, Germany). X-Ray Diffraction analysis was performed on ground powder samples in order to identify the phase composition (XRD, Miniflex 600, Rigaku, Japan) with copper Kα radiation. XRD analysis was achieved in the 2θ range of 20° and 50°. A quantitative analysis of the α:β phase ratio was made using the intensity of the (102) and (210) peaks of the α-SiAlON phase, and the (101) and (210) peaks of the β-SiAlON phase. This was performed using the equation presented below.

(2) $$\frac{I_{\beta }}{I_{\beta }+I_{\alpha }}=\frac{1}{1+K[\left(\frac{1}{w_{\beta }}\right)-1]},$$

where *I_α_* and *I_β_* are observed intensities of αı and βı-SiAlON peaks, respectively, *W_β_* is the relative weight fraction of βı-SiAlON, and *K* is the combined proportionality constant resulting from the constants in the two equations, namely:

(3) $$I_{\beta }=K_{\beta }*W_{\beta },$$

(4) $$I_{\alpha }=K_{\alpha }*W_{\alpha },$$

which is 0.518 for β (101) and α (102) reflections, and 0.544 for β (210) and α (210) reflections [31].

Room temperature dielectric properties of the starting powders were measured by the open-ended coaxial probe technique [32] in the 0.5–3 GHz frequency range, by using an Agilent 8753D network analyzer (Keysight, California, USA) connected to an Agilent 85070E dielectric kit probe (Keysight, California, USA). Measurements were performed placing the probe as the bottom support of a glass graduated tube, 20 mm in diameter, with side silicon-based O-rings to avoid powder leakage from the junction region. The powders were poured into the resulting container, reaching a reference level of 50 mm, then mechanically tapped until a constant volume was achieved. This approach allows to qualitatively compare the measured dielectric properties under similar density and probe contacting conditions. At least three measurements were performed for each sample.

## 3. Results and Discussion

### 3.1. Permittivity Measurement of the Starting Compositions

The 2.45 GHz room temperature dielectric properties of the loose powders are reported in Table 3. Values differ significantly from the ones used in the model, which were referred to as sintered SiAlON, as they take into account the presence of porosity (void spaces between particles), as a result of the sample preparation procedure previously described. From the tan δ values, it can be easily observed how the addition of small percentages of TiN to the initial powder mixture significantly contributes to increasing the microwave absorbencyof the specimen to be sintered. 

### 3.2. Phase and Microstructural Analysis

**Figure 8:**
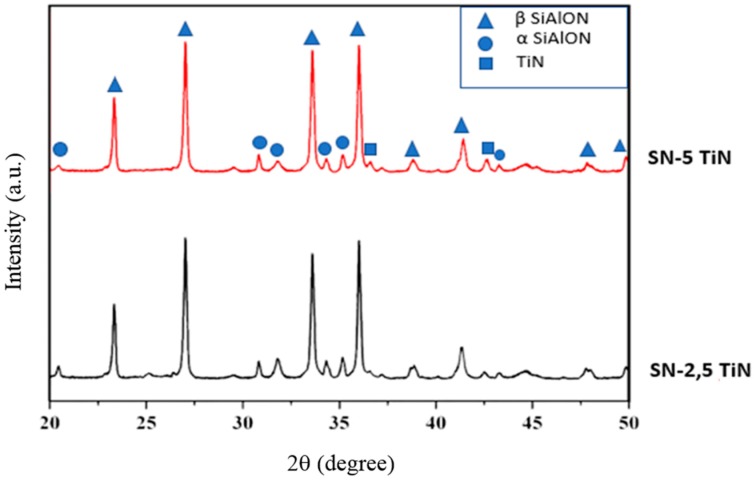
X-Ray Diffraction (XRD) analysis of the microwave sintered SN-TiN composites.

The XRD analysis results shown in Figure 8 demonstrate that there were no new phases detected apart from α-SiAlON (PDF-JCPDS 42-0251), β-SiAlON (PDF-JCPDS 48–1615), and TiN (PDF-JCPDS 38-1420). According to the α:β ratio calculations, the highest amount of β-SiAlON was obtained in the sample with 5 wt.% TiN which contains 26% α- and 74% β-SiAlON, whereas the sample with 2.5 wt.% TiN additive contains 31% α- and 69% β-SiAlON.

**Figure 9:**
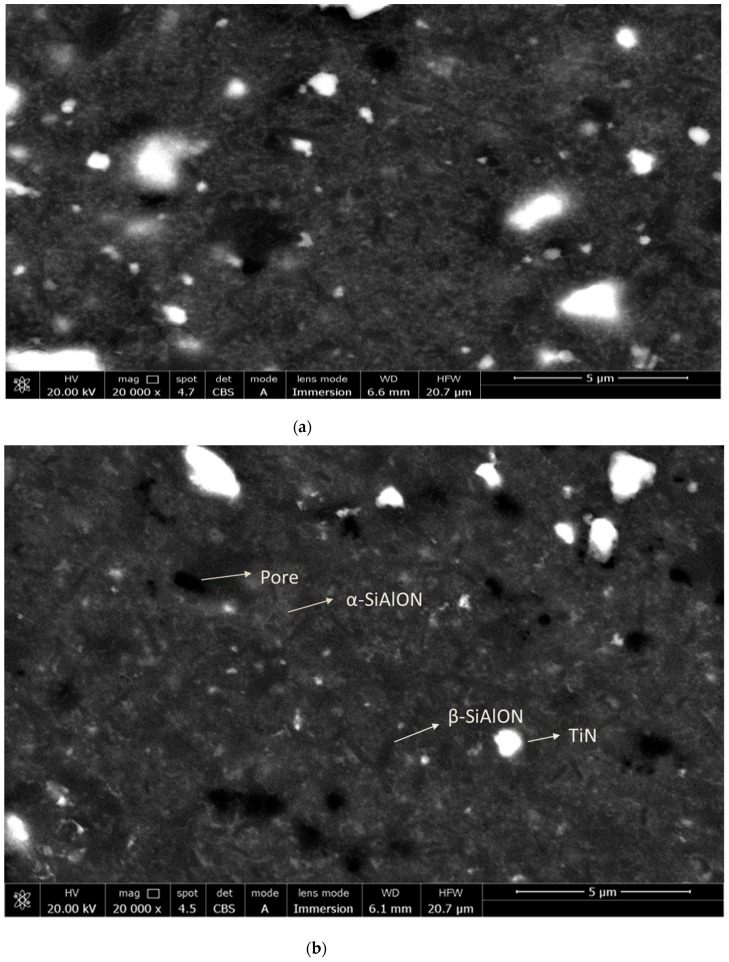
SEM micrographs of SN-TiN composites of (**a**) SN-2.5 TiN sintered at 780 °C and (**b**) SN-5 TiN sintered at 850 °C.

Typical back-scattered SEM images of the final microstructures achieved from the polished cross sections of SN-2.5 TiN and SN-5 TiN samples are given in Figure 9, where various phases can be distinguished due to sufficient atomic number contrast; the β-SiAlON grains (which contain no rare earth elements) are darker and more needle-like, whereas the α-SiAlON grains (which contain a small amount of rare earth elements) are gray and equiaxed, whilst rare earth-rich grain boundary phase appears white. TiN particles were distributed homogeneously in the microstructure and protected their angled structure. Formation of thin elongated β-SiAlON grains was observed in the microstructure due to the low sintering temperature and short sintering time. Additionally, well dispersed but fine α-SiAlON grains were located between β-SiAlON grains. Some round shaped small pores were also observable. 

The small amount of β phase in the samples is probably due to the uncomplete α → β transformation in microwave sintered samples due to the short sintering time. Microwave sintered SiAlON samples containing 5 wt.% TiN developed a distinctly narrow grain-size distribution.

During the early stages of microwave sintering, a controlled heating rate is achieved; when the sample surface temperature reaches 500 °C, the heating rate is rapidly increased, accompanied by a decrease of the amount of reflected power. This suggests that the sample is becoming lossier and possible thermal runaway is occurring as well. This phenomenon is most likely ascribable to the coupling of microwave power with the dielectrically lossier grain boundary glassy phase, which is volumetrically heated. Despite the impossibility to perform high temperature dielectric properties measurement of this glassy phase, it is very likely that it is absorbing microwaves, as typically encountered in the literature [33]. This promotes the dissolution of α–SiAlON and nucleation of β-SiAlON. 

The sintered samples presented a “reverse” density gradient (i.e., opposite to what one expects from conventionally sintered ceramics) in the upper portion of the samples, suggesting that a reverse temperature gradient existed during microwave sintering, as also evidenced from the modeling stage. This is ascribed to the heat losses from the sample upper surface, not in contact with the auxiliary SiC absorber, nor with the alumina lining, while heat is generated volumetrically. This phenomenon could be reduced using a different auxiliary absorbers setup and reducing the heating rates. The reverse density gradient results in a transition from open porosity at the sample surface to closed porosity in the immediately inner layers, of which the measurements of Table 4 are referred to. 

Despite an almost identical green density, the addition of larger quantities of TiN resulted in a high sintered density. Considering that literature results on microwave sintering of α-β SiAlON reports, during the densification stage, an average density variation of 0.072%K^−1^ [34], the measured temperature difference between the two sets of samples can be considered responsible for a percentage of densification in excess of 5%. Hence, a specific effect of the TiN addition alone on densification cannot be concluded. However, TiN additions in the SiAlON system, together with a higher sintering temperature, promoted densification during microwave sintering. However, densification appears to be limited in microwave sintered samples as surface porosity results from selective localized overheating and decomposition of TiN-containing phases [20,35]. 

### 3.3. Mechanical Properties

The Vickers Hardness and Vickers Indentation Fracture Toughness of the microwave sintered composites are presented in the Table 5. Mechanical properties of α–β SiAlON ceramics depend on the relative amounts, size and shape of the α- and β-SiAlON phases, in a similar manner to the Si_3_N_4_ ceramics. α-SiAlON features are typically equiaxed in shape and the hardness is improved by this feature due to the cations present in a lattice structure such as Ca [36]. However, simultaneously as a result of the shape factor, the material’s fracture toughness is reduced. β-SiAlON grains tend to be elongated in shape which acts similar to a whisker and thus increases the fracture toughness. It is possible to produce both hard and tough SiAlON ceramics by stabilizing both α and β structures in the same microstructure [37]. The SN-5 TiN sample has a higher hardness value although it has a lower α-SiAlON content than the sample SN-2.5 TiN. In this case, the relative densities of the samples take importance as the sample SN-5 TiN has higher relative density than the sample SN-2.5 TiN. 

## 4. Conclusions 

α/β-SiAlON-TiN composites were successfully sintered in the single mode microwave furnace operating at 2.45 GHz frequency with a power output of 660 W. The Finite-Difference Time-Domain (FDTD) software was found very useful in assessing the most suitable experimental setup. Dielectric measurement results showed that even a small addition of TiN enhanced the microwave absorbing properties of SiAlONs. The highest relative density of 96% and mechanical properties (Vickers hardness: 12.98 ± 1.81 GPa and Vickers indentation fracture toughness: 5.52 ± 0.71 MPa.m^1/2^) were obtained in the sample which contains 5 wt % TiN with 30 min of sintering time at 850 °C. According to SEM, fine grain size was obtained due to the fast microwave sintering nature. Final products consist of α-SiAlON, β-SiAlON and TiN crystalline phases. α:β ratios showed that the sample SN-5 TiN was very close to the to the desired composition of 25% α:75% β. 

## Figures and Tables

**Table 1:** Material parameters used in the simulation.

Material	Density (g/cm^3^)	Permittivity		Thermal Conductivity (W/mK)		Specific Heat (J/gK)	
		T = 25 °C	T = 1000 °C	T = 25 °C	T = 1000 °C	T = 25 °C	T = 1000 °C
**Pressed powders**	3.25	7.6 − i × 0.023	9.5 − i × 0.376	14	835	0.620	1.210
**SiC**	3.21	9.2 − i × 1.60	7.3 − i × 0.25	280	90	0.6	1.2
**Alumina fiber**	4.1	1.39 − i × 0.001	1.6 − i × 0.03	0.06	0.2	0.764	1.258
**Air**	0.001225	1	1	0.0255	0.080	1.007	1.180

**Table 2:** Composition and sintering temperature of the microwave sintered samples.

Sample Code	Composition	Max Sintering Temperature (°C)
SN-2.5 TiN	25α:75βSiAlON-2.5 wt.%TiN	780
SN-5 TiN	25α:75βSiAlON-5 wt.%TiN	850

**Table 3:** Dielectric properties of the starting powders at 2.45 GHz frequency at room temperature. (Average of three different measurements performed on each starting composition).

Sample Code	Real (ε′)	Imaginary (ε″)	Loss Tangent (tgδ) = (ε″/ε′)
25α:75β-SiAlON (SN)	1.63 ± 0.01	0.01 ± 0.001	0.006
SN-2.5 TiN	1.92 ± 0.01	0.15 ± 0.001	0.08
SN-5 TiN	2.02 ± 0.01	0.18 ± 0.001	0.09

**Table 4:** Sintering time, sintering temperature and relative densities of the microwave processed samples.

Sample Code	Sintering Time (h)	Sintering Temperature (°C)	Relative Density (%)
SN-2.5 TiN	0.5	780	91
SN-5 TiN	0.5	850	96

**Table 5:** Vickers hardness and Vickers Indentation fracture toughness of the sintered samples.

Sample Code	Sintering Time (h)	Vickers Hardness (GPa)	Vickers Indentation Fracture Toughness, VIF (MPa.m^1/2^)
SN-2.5 TiN	0.5	9.31 ± 0.79	3.96 ± 0.57
SN-5 TiN	0.5	12.98 ± 1.81	5.52 ± 0.71
